# An Integrated View of Whole-Tree Hydraulic Architecture. Does Stomatal or Hydraulic Conductance Determine Whole Tree Transpiration?

**DOI:** 10.1371/journal.pone.0155246

**Published:** 2016-05-25

**Authors:** Juan Rodríguez-Gamir, Eduardo Primo-Millo, María Ángeles Forner-Giner

**Affiliations:** Department of Citriculture and Vegetal Production, Valencian Institute of Agrarias Research, Moncada, Valencia, Spain; University of Vigo, SPAIN

## Abstract

Hydraulic conductance exerts a strong influence on many aspects of plant physiology, namely: transpiration, CO_2_ assimilation, growth, productivity or stress response. However we lack full understanding of the contribution of root or shoot water transport capacity to the total water balance, something which is difficult to study in trees. Here we tested the hypothesis that whole plant hydraulic conductance modulates plant transpiration using two different seedlings of citrus rootstocks, *Poncirus trifoliata* (L.) Raf. and Cleopatra mandarin (*Citrus reshni* Hort ex Tan.). The two genotypes presented important differences in their root or shoot hydraulic conductance contribution to whole plant hydraulic conductance but, even so, water balance proved highly dependent on whole plant conductance. Further, we propose there is a possible equilibrium between root and shoot hydraulic conductance, similar to that between shoot and root biomass production, which could be related with xylem anatomy.

## Introduction

Transpiration in plants plays a critical role in plant physiological processes, affecting carbon uptake by leaves, growth and productivity and depends on plant hydraulics, stomatal conductance and/or environment conditions [1, 2, 3, 4]. Plant hydraulics constrains ecosystem productivity by setting physical limits to water transport [5, 6], thus its study is essential to understanding plant water-use regulation and its associated impact on water balance.

Hydraulic conductance can be variable throughout the day [7] or in response to environmental conditions [8, 9]. Besides, maximum transpiration rates have been related to maximum values of whole plant hydraulic conductance [10]. Studies suggest that hydraulic conductance of different plant organs can regulate the opening and closing of stomata in response to vapor pressure deficits [11, 12], regulating, thus, plant water relations. In this respect, there are existing studies that link hydraulic conductance of leaf [13, 14, 15, 16, 17, 18, 19], shoot [20], stems [21, 22] or root [23, 24, 25] with transpiration or stomatal conductance. However, there is a lack of understanding about the integration of whole woody plant hydraulics and its implication in water relations. As [26] state, few studies have examined all the organ components of the hydraulic pathway to evaluate how they relate to whole plant conductance and most work on hydraulic transport of woody plants has been conducted on lateral stems or branches. Regarding different organ contribution, [11, 27, 28] propose that approximately 50–60% of the whole-plant hydraulic resistances are located in the root system. [10] found higher values of shoot hydraulic conductance compared with root hydraulic conductance values in sunflower. However on kiwifruit plants, root hydraulic conductance was higher than shoot hydraulic conductance [29]. It is evident that there is inconsistent information on the contribution of root or shoot hydraulic conductance to total hydraulic conductance and, more so, to plant transpiration.

Most studies on whole plant hydraulic architecture parameters with exchange parameters use the evaporative flux method [30]. This method assumes the hydraulic conductance of the soil/root/leaf pathway is *K* = *E*/Δ*p* (Kg s^-1^ MPa^-1^), where Δ*p* is the difference in the water potential between the two considered points (e.g. between soil water potential and leaf water potential), and *E* is the amount of water lost through transpiration at the time plant water potential is determined. The method has been validated [31], however, it makes possible to establish better relationships between gas exchange parameters and hydraulic conductance. In our study, we measured hydraulic conductance with a high-pressure flow meter (HPFM), therefore hydraulic trait measurements were totally independent of gas exchange measurements.

Furthermore, plant hydraulics is a complex system and its regulation is complex as well. It can depend on root anatomy, interactions between water and solute flow or on aquaporin activity in cell membranes [9, 32, 33, 34]. Many studies indicate stem or root hydraulic conductance in trees are related with xylem structure [35, 36]. And also, plants have the ability to adjust their water uptake capacity to changing environmental conditions by regulating aquaporins in the plasma membrane [37].

Taking all this into account, we hypothesized that whole plant hydraulic conductance modulates plant transpiration. Accordingly, we tested whether plants that differ in their contribution of root and shoot hydraulic conductance to whole plant hydraulic conductance also differ in their hydraulic conductance-transpiration relationship. We also studied the influence of xylem anatomy on root and shoot hydraulic conductance, as well as its possible effect on different plant-part contribution to plant hydraulic conductance.

To do so, we used seedlings of two different citrus rootstocks *Poncirus trifoliata* (L.) Raf. (PT) and Cleopatra mandarin (*Citrus reshni* Hort ex Tan.) (CM). Citrus rootstocks present different hydraulic characteristics that determine tree behavior, water relations or response to different stresses. *Poncirus trifoliata* and CM differ greatly at both physiological and morphological levels. Cleopatra mandarin is characterized by simple leaves versus PT that presents trifoliate leaves, smaller than those of CM. In previous studies, PT showed higher transpiration rates than CM, both in seedlings and in varieties grafted on them [23, 38, 39], and higher root hydraulic conductance values than CM [23, 38, 40]. To date studies of shoot hydraulic conductance of these genotypes are lacking.

## Material and Methods

### Plant Material and growth conditions

Ten-month-old seedlings of *Poncirus trifoliata* (PT) and Cleopatra mandarin (CM) were used in the experiment. PT and CM seeds were harvested from the mother seed trees held in the germplasm collection at IVIA, Valencia, Spain. Seeds were sown on 55x40 cm trays containing a mixture of peat and siliceous sand (3:2 v:v) in an aphid-proof greenhouse with a cooling system that kept temperatures between 15°C and 18°C and 80% relative humidity. Plants were grown with supplementary light (<50 μmol m^-2^ s^-1^, 400–700 nm) to extend the photoperiod to 16 h. Five-month-old plants were transplanted into 3 L pots with a mixture of peat and siliceous sand (3:2 v:v) and were watered twice a week with the following nutrient solution: 3 mMCa(NO_3_)_2_, 3 mM KNO_3_, 2 mM MgSO_4_, 2.3 mM H_3_PO_4_, 17.9 mM Fe-EDDHA and micronutrients as described by [41]. The pH of the nutrient solution was adjusted to 6.0 with 1 M KOH or 1 M H_2_SO_4_.

At the beginning of the experiment, sixteen plants of each genotype with no branches, (i.e., all leaves were on the main stem) and with different levels of development, were selected. Plants were selected for the experiment according to individual whole plant transpiration (measured gravimetrically before to the experiment, as described below) as we wanted to have a wide range of whole plant transpiration values and wanted this range to be similar for both genotypes. Selected CM plants had between 17 and 77 leaves and PT plants had 15 to 80 leaves. The experiments were conducted at the IVIA, Valencia, Spain (39.28 N—0.22 W) during late summer, with a photoperiod of 14 daylight hours. The plants were outdoors where average temperature was 24 ± 1°C and relative humidity, 80%.

During the experiment, plants were contained individually in the aforementioned 3 L pots and irrigated with 1.5 L water daily. The experiment lasted 5 days and plants were randomly distributed and surrounded by a guard row (buffer) not included in the experiment.

### Whole plant transpiration (*T*_p_)

From day 1 to 3, to measure whole plant transpiration, after each irrigation pots were covered with a sheet of plastic with a hole of similar diameter to that of the plant stem, through which the plant protruded. This system prevented evaporation from the substrate. The daily transpiration of each plant (*T*_p_) was calculated as the difference between the weight of the watered pot (after draining) and the weight of the pot before watering the following day. The mean of three determinations (one per day) on each plant was considered as representative of each individual plant

### Gas exchange and vapor pressure deficit

Transpiration rate (*E*, *mmolH*_*2*_*O m*^*-2*^ *s*^*-1*^) and stomatal conductance (*g*_s_, *mmolH*_*2*_*Om*^*-2*^ *s*^*-1*^) were measured on days 2 and 3, recording conditions of ambient light, temperature, relative humidity and CO_2_ concentration with an LCi Portable Photosynthesis System (ADC, herts, UK). The measurements were taken from 8:00 am to 6:00 pm. Air and leaf temperatures and photosynthetic photon flux density, provided by the LCi ranged from 19.1 to 32.2°C and 101.5 to 1971.7 μmol m^-2^ s^-1^, respectively. Both parameters reached their maximum values between 10:00 am and 3:00 pm. The *E* and *g*_s_ were measured in fully expanded leaves taken from the central part of each plant. At each sampling time, six different leaves were measured in six different plants for each type plant.

Leaf-to-air vapor pressure deficit (*VPD*) is given by *VPD* = e_s_−e_air_, where e_air_ is the partial pressure of water vapor in the air, while e_s_ is the saturation water vapour pressure as it is assumed that water vapor pressure in the substomatal cavity is close to 100% [42]. e_air_ was provided by the LCi. e_s_ was calculated by: e_s_ = 6,1373 10^−3^ e^(T_leaf_*(18,568-T_leaf_/254)/(T_leaf_+255,57)), obtained by the numerical integration of Clausius-Clapeyron equation, where e_s_ is the saturation water vapour pressure (bar) and T_leaf_ is the leaf temperature (°C) measured with the LCi_._

### Root, Shoot and Plant hydraulic conductance

On day 4 and 5 of the experiment, root hydraulic conductance (*K*_r_, *KgH*_*2*_*O MPa*^*-1*^ *s*^*-1*^) and shoot hydraulic conductance (*K*_s_, *KgH*_*2*_*O MPa*^*-1*^ *s*^*-1*^) were measured in each experimental plant with a High Pressure Flow Meter (HPFM) (Dynamax Inc., Houston, TX). We followed the method described by [43]. To minimize the potential impact of diurnal periodicity on hydraulic conductance [10], all measurements were taken between 10:00 am and 2:00 pm at ambient temperature (24 ± 1°C).

The plants were cut 5 cm above the soil surface and the stumps of shoots and roots were connected to the HPFM with a water-tight seal, and the conductance of these plant parts (*K*_s_ and *K*_r_) was determined using the transient measurement mode. Plant hydraulic conductance (*K*_p_) was calculated by the equation 1/*K*_p_ = 1/*K*_r_+1/*K*_s_.

At the end of the measurements, the leaf areas of each plant were measured with a Li-Cor Li-3100 Area Meter (Li-Cor, inc. Lincoln, Nebraska, USA). All plant fractions were dried in a forced-draft oven at 60°C for 48 hours and weighed, and we calculated the Leaf dry weight/Root dry weight ratio (L/R).

We calculated leaf-specific root hydraulic conductance (*K*_r-l_) and leaf-specific shoot hydraulic conductance (*K*_s-l_) by dividing *K*_r_ or *K*_s_, respectively, by the total plant leaf area. Root biomass-specific root hydraulic conductance (*K*_r-r_) was calculated by dividing *K*_r_ by the root dry weight.

### Light microscopy

Samples of about 3 mm of pioneer roots and 3 × 2 mm of wood from basal stem and taproot wood from seedlings were fixed, dehydrated and embedded in LR White (London Resin Co., Woking, Surrey, UK) according to [44]. Semi-thin transverse sections (1 μm thickness) were cut with a Leica RM2165 Rotary Microtome (Leica Instruments, Heidelberg, Germany) and stained with toluidine blue 0 (CI 52040, Merck, Darmstad, Germany) according to O’Brien et al. (1964). Representative sections of two tissue samples per plant and organ part (basal stem and taproot wood) from six independent plants of each seedling were examined and photographed with an Olympus BX-51 microscope (Olympus Imaging Corp., Tokyo, Japan), with a digital camera DP-12 and the Analysis program (Soft Imaging System GmbH, Munster, Germany). The anatomical data correspond to the mean of six independent plants on each rootstock. Three samples per tissue and plant were studied, and the average values were considered as representative of each individual plant. For each sample, values are the mean of three visual fields from three sections.

### Statistical Analyses

Although plant development differed, data for biomass, whole plant transpiration and hydraulic conductance showed a normal distribution. Therefore, in a first analysis, parameters were tested by analysis of variance (ANOVA) and comparisons of means were determined by the least significant differences (LSD) method, at 95% confidence level with Statgraphics Plus ver. 5.1 (Statistical Graphics, Englewood Cliffs, NJ, USA). Additionally, slopes and intercepts of the performed linear regressions were compared between genotypes using the same software application.

## Results

### Plant biomass and whole plant transpiration

**Fig 1:**
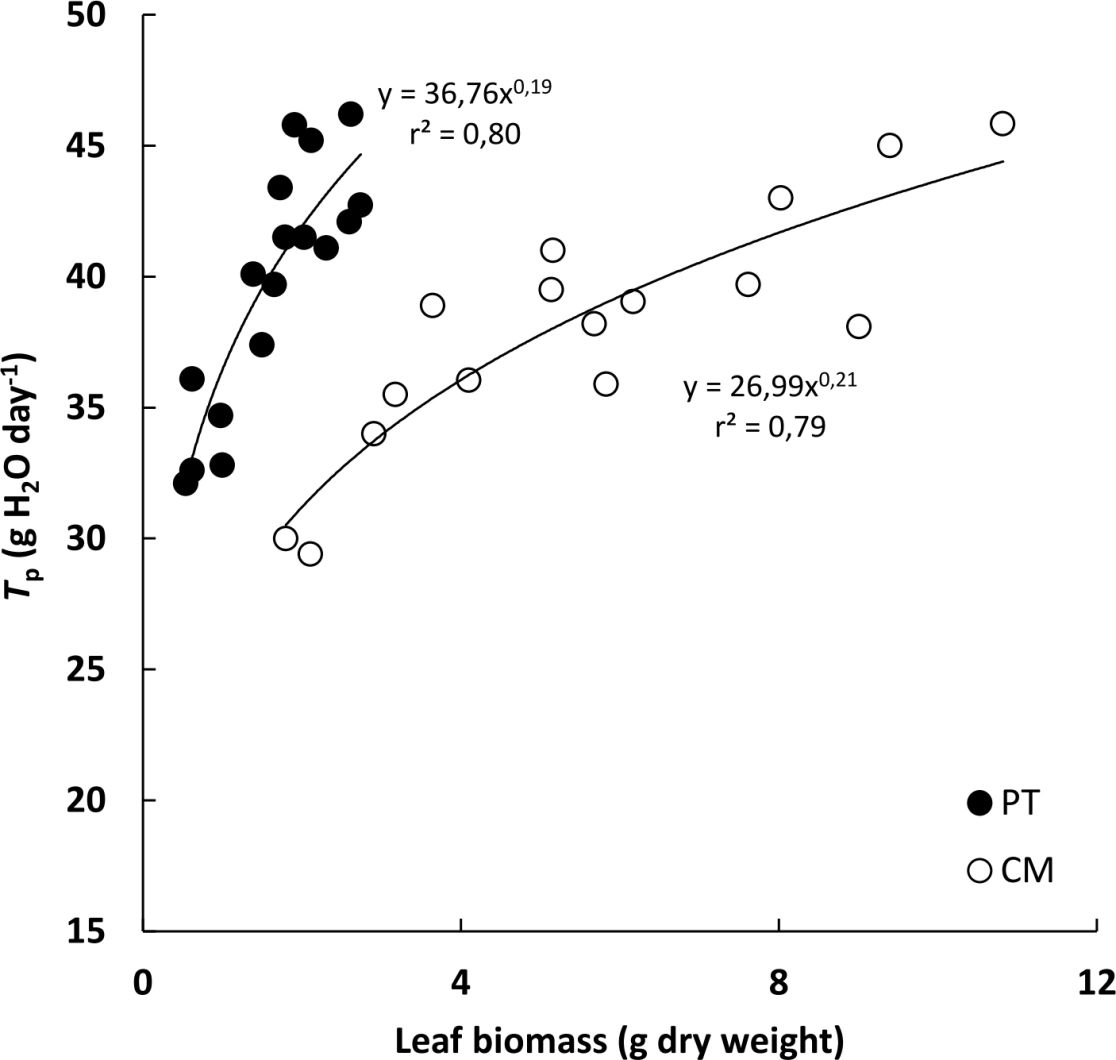
Relationship between foliar biomass and whole plant transpiration (*T*_p_) in *P*. *trifoliata* (PT) and Cleopatra mandarin (CM). Each point represents the mean of three measurements of *T*_p_.

We found morphological differences between the two types of plant (Table 1). This was due, in part, to the fact that Cleopatra mandarin (CM) is characterized by simple leaves while *P*. *trifoliata* (PT) presents smaller trifoliate leaves. Both genotypes were similar in terms of root system development. However, significant differences in foliar biomass values were found, being 3.65-fold higher for CM than for PT plants, which increased the Leaf dry weight/Root dry weight ratio (L/R) for CM compared with PT (3.72-fold higher in CM). Although CM had a higher foliar biomass than PT, whole plant transpiration (*T*_p_) values were similar in both plant groups. There was a positive and curvilinear correlation between leaf biomass and *T*_p_ for both genotypes (Fig 1).

**Table 1:** Dry weight of leaf (DW leaves) and roots (DW roots), leaf dry weight/root dry weight ratio (L/R), and whole plant transpiration (*T*_p_) in *P*. *trifoliata* (PT) and Cleopatra mandarin (CM).

	DW Leaves	DW Roots	L/R	*T*_p_
	(g)	(g)		g d^-1^
PT	1.55 ± 0.19 b	3.70 ± 0.31 a	0.44 ± 0.05 b	39.71 ± 1.20 a
CM	5.66 ± 0.68 a	3.68 ± 0.46 a	1.65 ± 0.15 a	38.07 ± 1.16 a

For each parameter, means (n = 16) with different letters show statistically significant differences (P<0.05).

### Gas exchange and vapor pressure deficit

**Fig 2:**
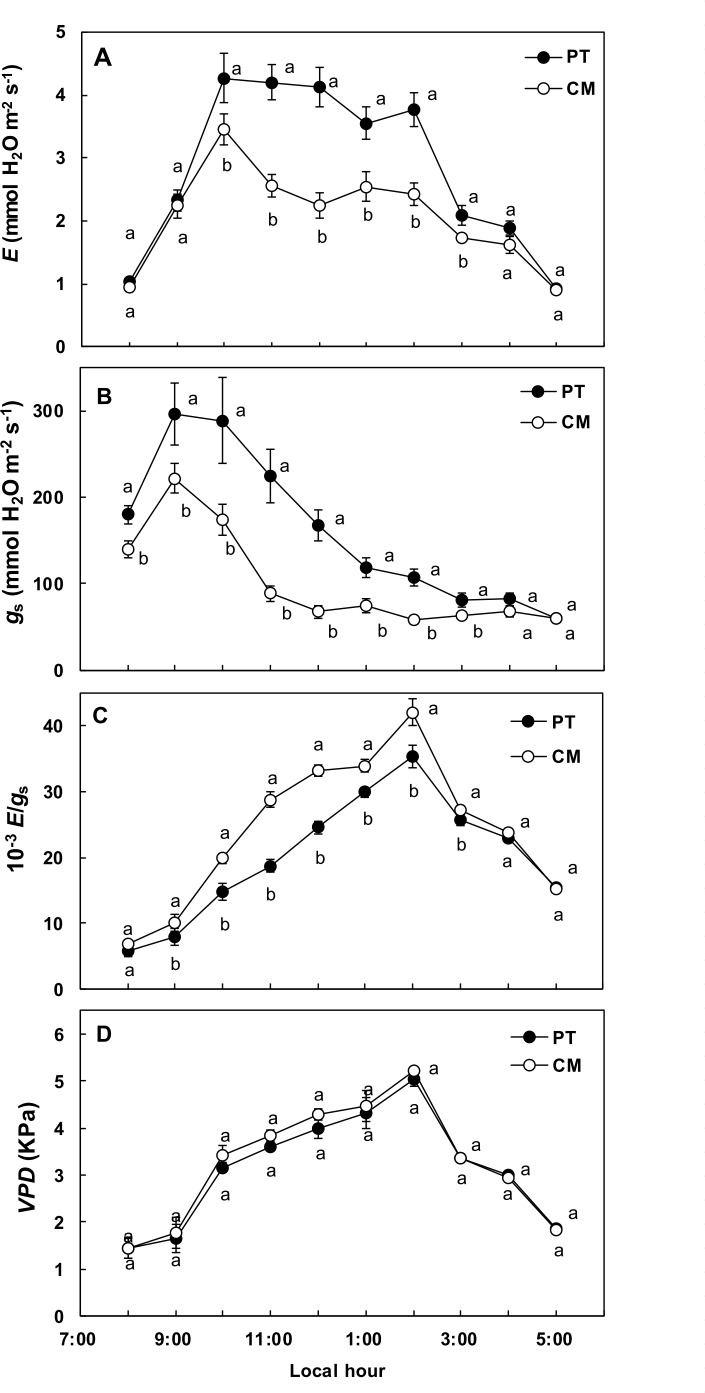
Diurnal time courses of stomatal conductance (*g*_s_), transpiration (*E*), *E*/*g*_s_ ratio and vapor pressure deficit (*VPD*) in *P*. *trifoliata* (PT) and Cleopatra mandarin (CM). Values are means of six replicates ± SE (n = 6). For each time, different letters indicate statistically significant differences (P <0.05) (LSD test).

Fig 2A shows the diurnal variations of transpiration rate (*E*) in leaves from plants of the two genotypes. Transpiration rate displayed typical behavior, increasing in the early morning (8:00 to 10:00 am) and then decreasing after 2:00 pm. Leaves from PT showed significantly higher *E* values throughout the day than leaves from CM (average *E* value between 10:00 am and 2:00 pm was 3.98 mmol H_2_O m^-2^ s^-1^ for PT and 2.68 mmol H_2_O m^-2^ s^-1^ for CM). Stomatal conductance (*g*_s_) (Fig 2B) differed with respect to *E* trend. Maximum *g*_s_ values were reached at 9:00 am in both genotypes and stomatal closure occurred from 10:00 am. CM presented significantly lower *g*_s_ values than PT and there was not a constant relationship between *E* and *g*_s_ during the day (ratio *E*/*g*_s_) (Fig 2C), which also differed between genotypes. CM had higher *E*/*g*_s_ values than PT between 9.00 am and 3.00 pm, despite the vapor pressure deficit (*VPD*), driving force for water loss by via transpiration, showed a similar pattern throughout the day for both genotypes with no statistical differences between them (Fig 2D). This suggests transpiration was not solely regulated by stomatal conductance.

### Root, shoot and plant hydraulic conductance

**Fig 3:**
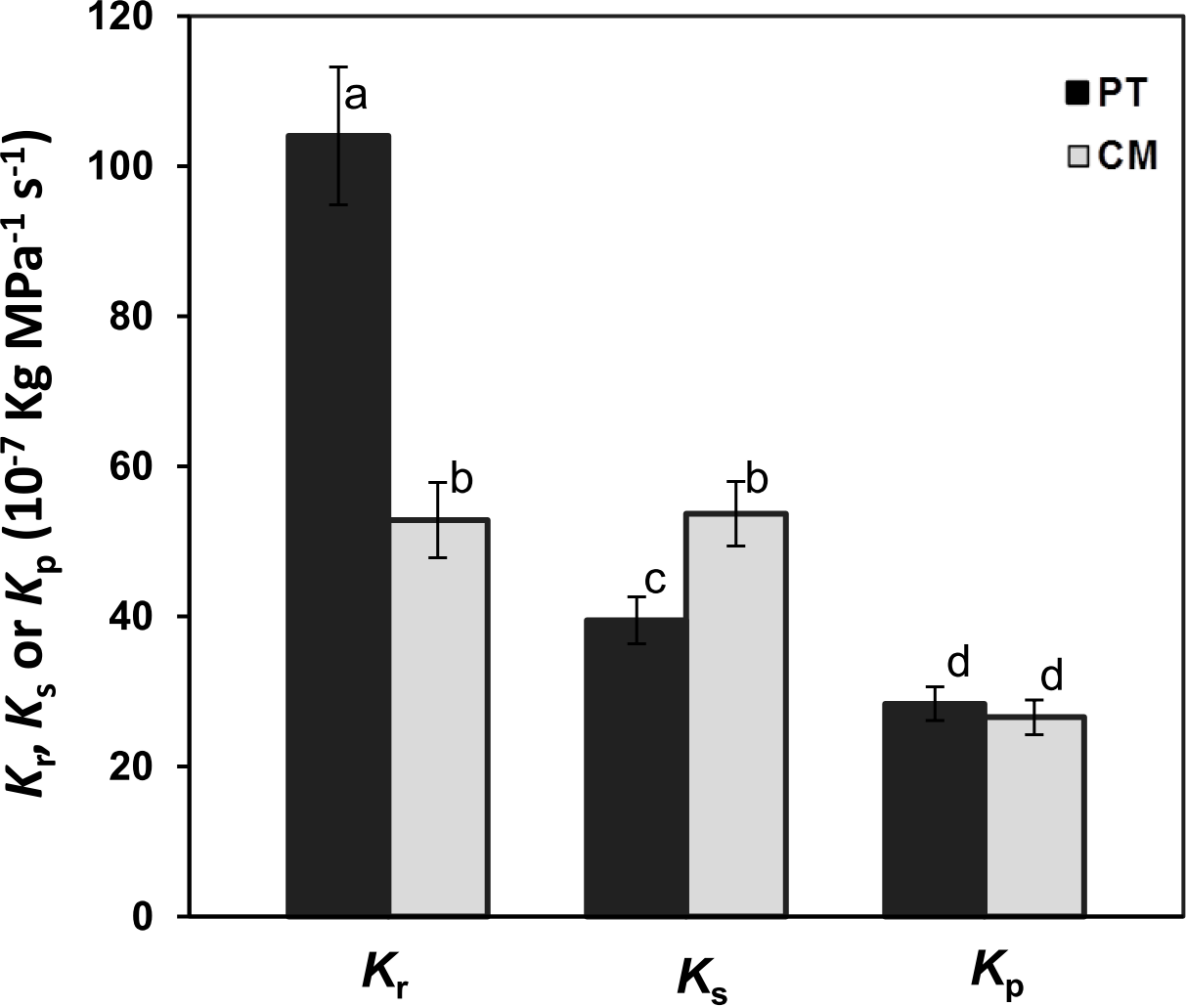
Root, shoot and plant hydraulic conductance (*K*_r_, *K*_s_ and *K*_p_, respectively), of *P*. *trifoliata* (PT) and Cleopatra mandarin (CM). The mean (n = 16) with different letters show statistically significant differences (P <0.05).

The average value of root hydraulic conductance (*K*_r_) was significantly higher in PT than in CM (104.03 10^−7^ and 52.87 10^−7^ Kg MPa^-1^ s^-1^, respectively) (Fig 3). This, together with the results presented in Table 1, which show a similar root-system biomass for both genotypes, demonstrates the higher water transport capacity of PT than CM at similar root biomass levels. However, shoot hydraulic conductance (*K*_s_) was significantly higher in CM than in PT (53.69 10^−7^ and 39,50 10^−7^ Kg MPa^-1^ s^-1^, respectively). Moreover, in PT seedlings, *K*_r_ was significantly higher than *K*_s_, while CM presented no significant differences between *K*_r_ and *K*_s_. Despite differences between *K*_r_ and *K*_s_ values between CM and PT, whole plant hydraulic conductance (*K*_p_) was similar in both plant types.

**Fig 4:**
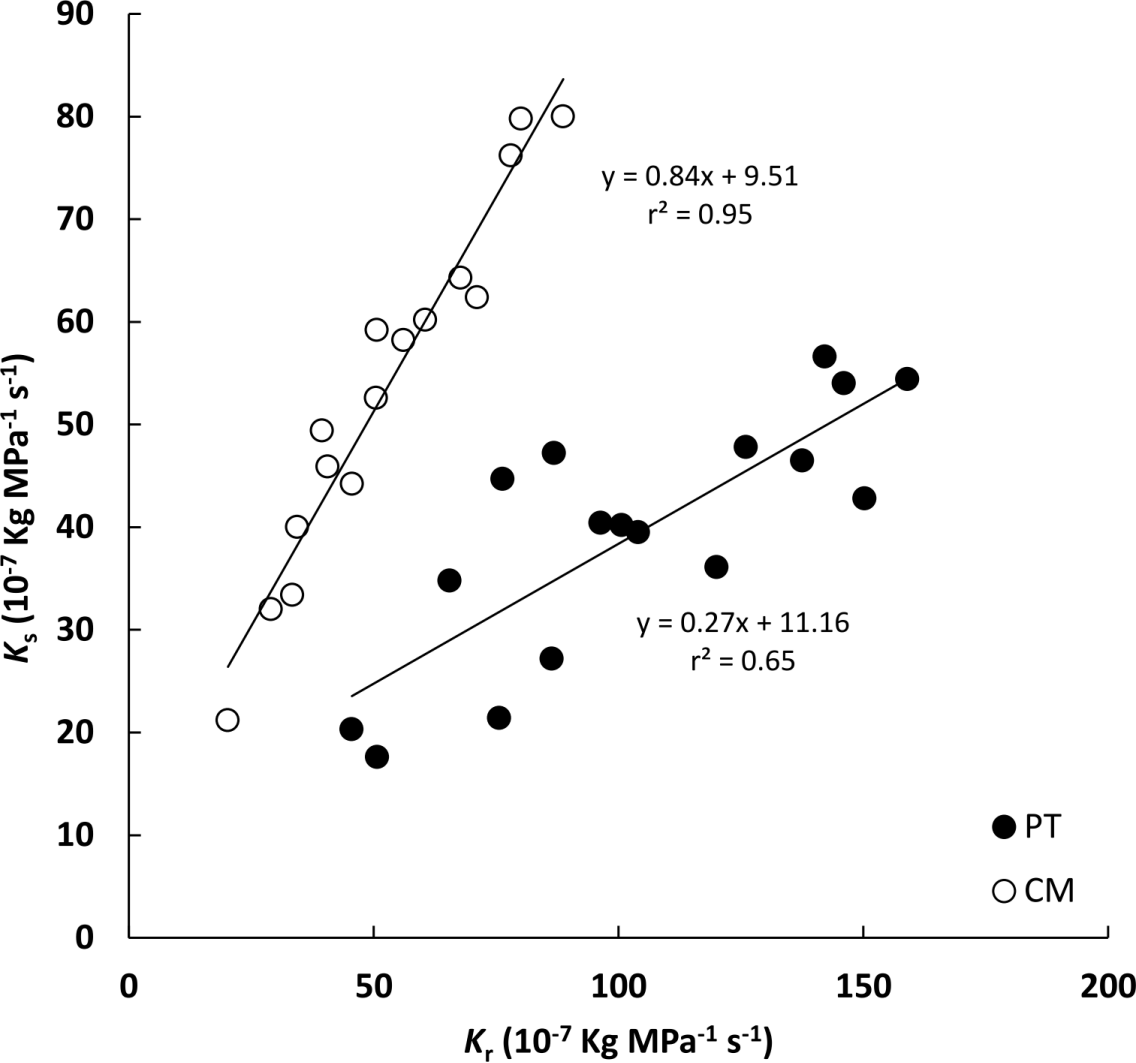
Relationship between root hydraulic conductance (*K*_r_) and shoot hydraulic conductance (*K*_s_) in *P*. *trifoliata* (PT) and Cleopatra mandarin (CM).

When the *K*_s_ value of each individual plant was plotted versus its respective *K*_r_ value, these two parameters were linearly related in both genotypes (r^2^ = 0.95 for CM, and r^2^ = 0.65 for PT) (Fig 4). However this relationship differed in each plant type. For a given *K*_r_ value, *K*_s_ was higher in CM than in PT plants.

### Relationship between hydraulic conductance and plant biomass

**Fig 5:**
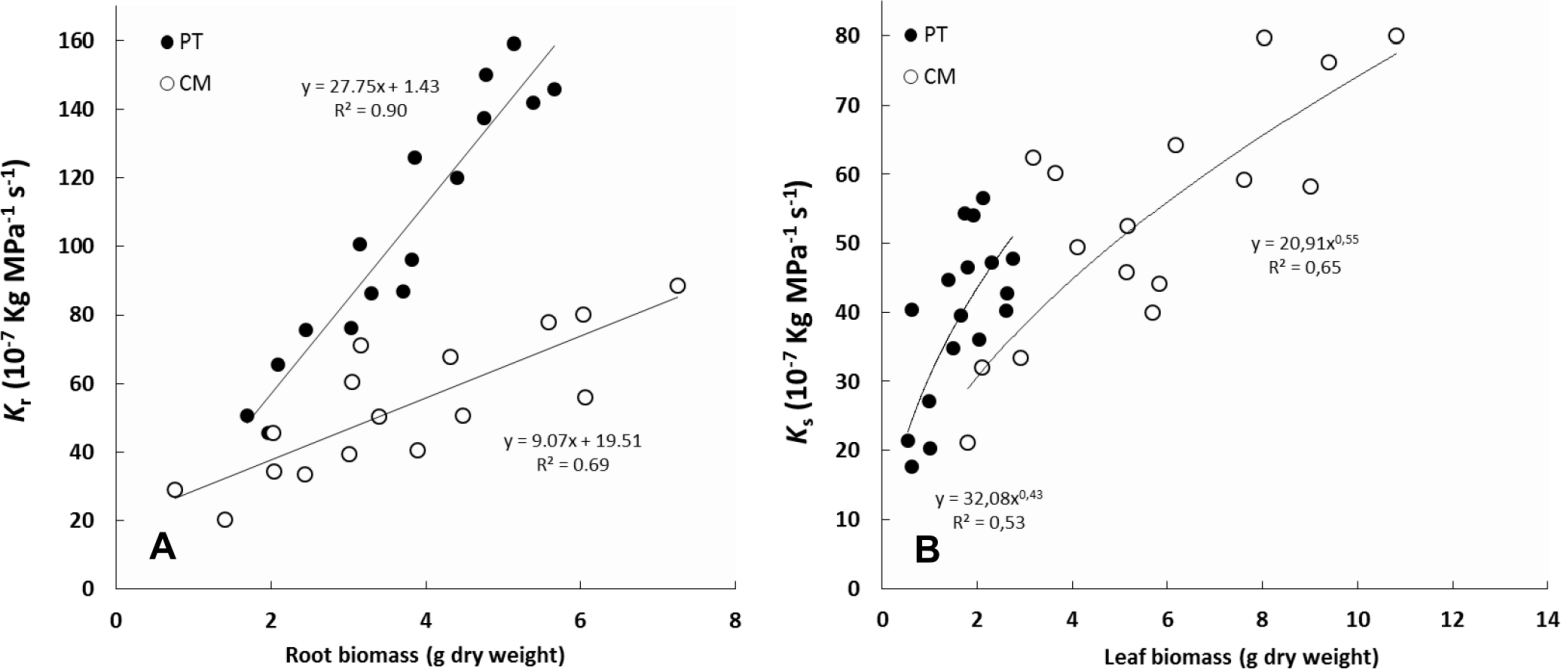
Relationship between root hydraulic conductance (*K*_r_) and root biomass and between shoot hydraulic conductance (*K*_s_) and the leave biomass expressed in g of dry weight in *P*. *trifoliata* (PT) and Cleopatra mandarin (CM).

In PT, root hydraulic conductance values measured (*K*_r_) ranged from 45.60 10^−7^ to 159.00 10^−7^ Kg MPa^-1^ s^-1^ for roots which their dry weight ranged between 1.69 and 5.67 g dry weight (Fig 5A). In CM, *K*_r_ values ranged from 20.20 10^−7^ to 88.70 10^−7^ kg MPa^-1^ s^-1^ for roots between 0.76 and 7.25 g dry weight. *K*_r_ increased linearly both for CM and for PT with increased root biomass, with r^2^ values of 0.90 and 0.69 for PT and CM, respectively. For a given root biomass, *K*_r_ values were higher in PT than in CM.

Leaves of PT plants of the experiment showed values between 0.54 and 2.74 g dry weight (Fig 5B). *K*_s_ values of PT were between 17.60 10^−7^ and 56.60 10^−7^ Kg MPa^-1^ s^-1^. In CM, whose foliar biomass ranged from 1.80 to 10.82 g dry weight, *K*_s_ values were in a range 21.21 10^−7^ to 80.03 10^−7^ kg MPa^-1^ s^-1^. There was a curvilinear increase in *K*_s_ with increased foliar biomass presenting r^2^ values of 0.53 and 0.65 for PT and CM, respectively. For similar foliar biomass, PT presented higher *K*_s_ values.

### Relationship between whole plant transpiration and root, shoot and plant hydraulic conductance

**Fig 6:**
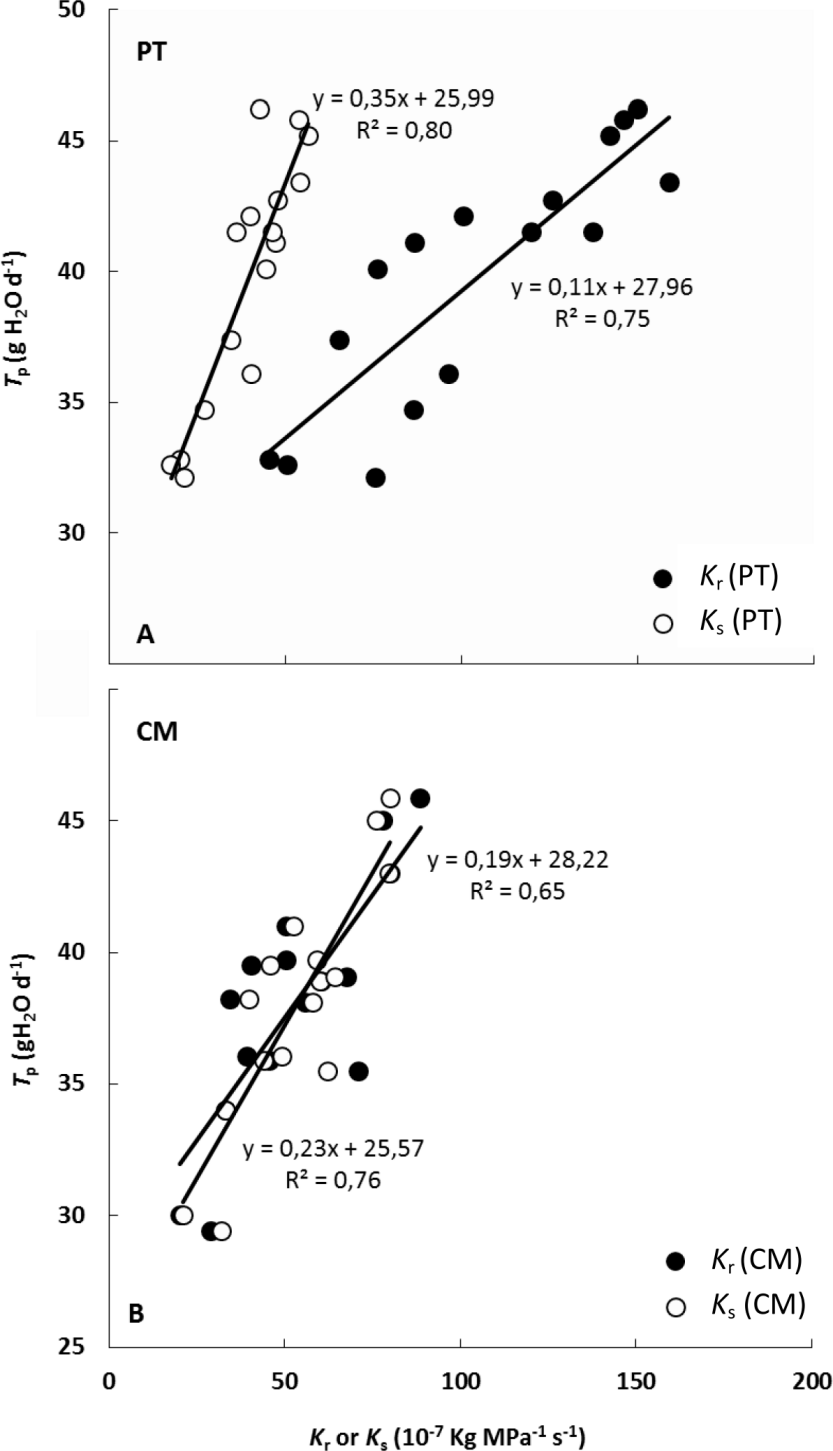
Relationship between root hydraulic conductance (*K*_r_) and shoot hydraulic conductance (*K*_s_) with whole plant transpiration (*T*_p_) in *P*. *trifoliata* (PT) and Cleopatra mandarin (CM). Each point represents the mean of three measurements of *T*_p_.

Whole plant transpiration (*T*_p_), measured gravimetrically, increased linearly with increasing *K*_r_ and *K*_s_ values in both genotypes (Fig 6). In CM, *T*_p_ was affected in a similar way by *K*_r_ and *K*_s_ (Fig 6B) (there were no differences in the slope and the ordinate of the regression line between both regression lines). However, in PT plants, there was a greater *T*_p_ increase when *K*_s_ rose than when *K*_r_ rose (Fig 6A).

**Fig 7:**
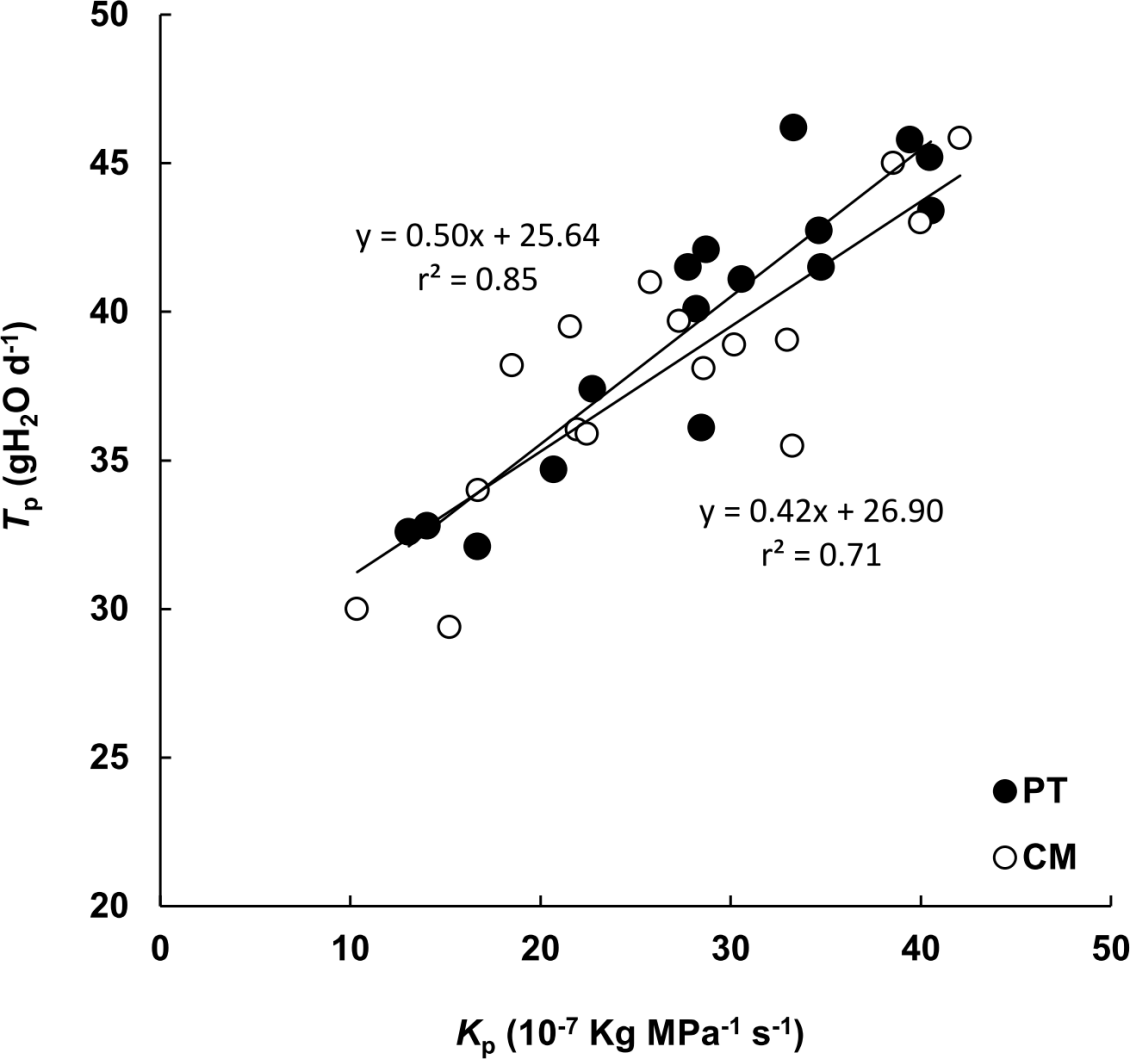
Relationship between plant hydraulic conductance (*K*_p_) and whole plant transpiration (*T*_p_) in *P*. *trifoliata* (PT) and Cleopatra mandarin (CM) in sixteen independent plants for each seedling. Each point represents the mean of three measurements of *T*_p_ for each plant.

Moreover, the hydraulic conductance of the whole plant (*K*_p_) was linearly related to *T*_p_ (Fig 7). This relationship was identical in both genotypes (slopes and intercepts were not significantly different) despite morphological, physiological and hydraulic traits these genotypes presented.

### Xylem anatomy

**Fig 8:**
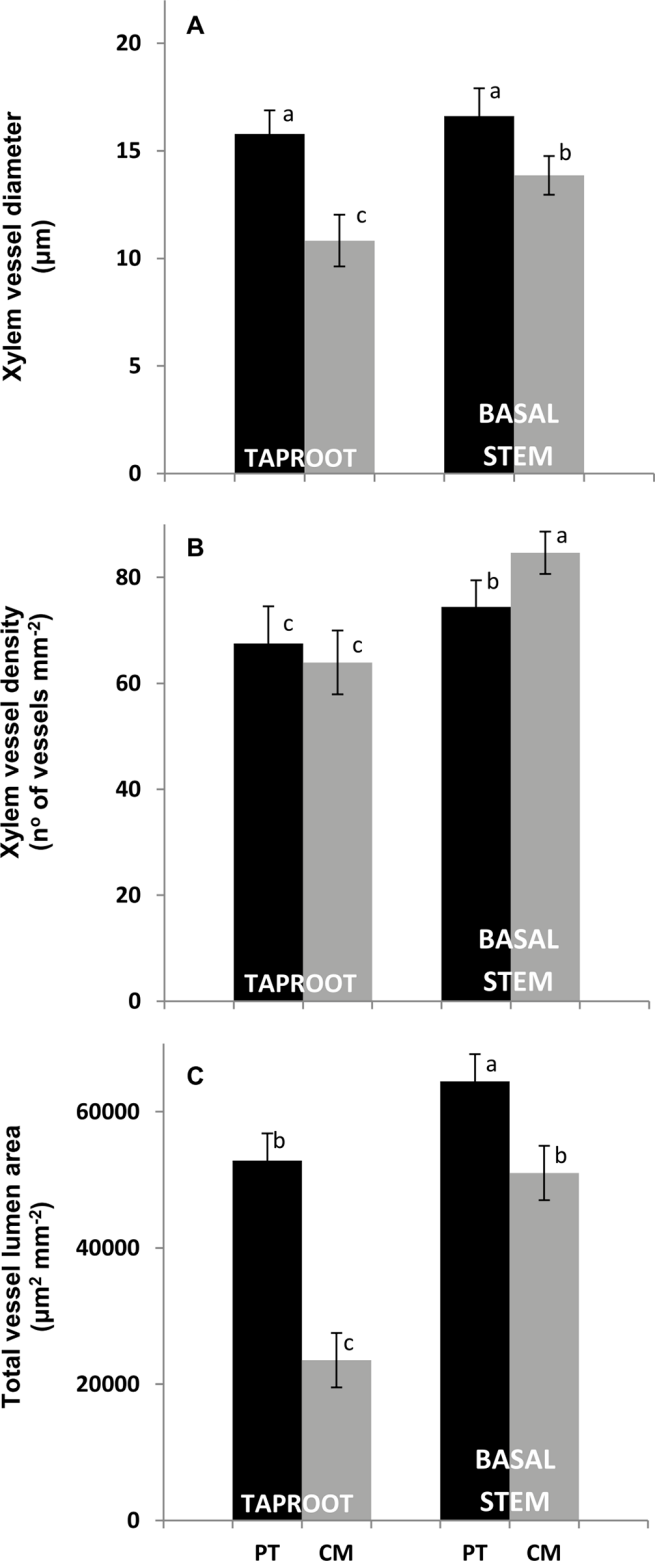
(A) Diameter, (B) density and (C) total lumen area of xylem vessels of taproot and basal stem in cross sections of *Poncirus trifoliata* (PT) and Cleopatra mandarin (CM) seedlings. Histological data correspond to the mean of six independent plants (n = 6) of each rootstock. The value for each plant is the mean of three visual fields of three sections from three samples per root and stem. Different letters indicate statistically significant differences (P <0.05) (LSD test).

**Fig 9:**
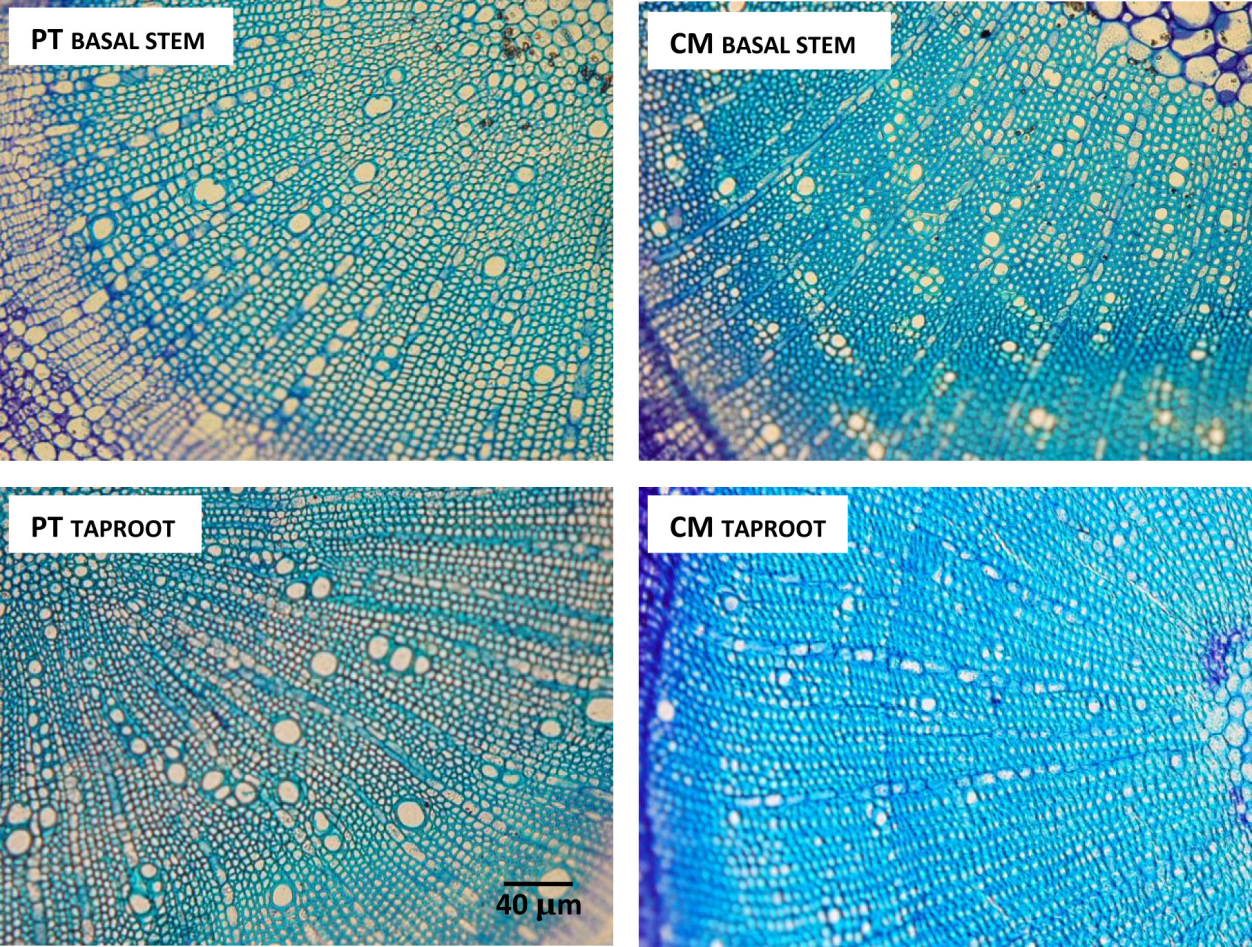
Light micrograph of secondary xylem from basal stem and taproot of *P*. *trifoliata* (PT) and Cleopatra mandarin (CM). Sections were cut at 20–25 mm under and above to soil surface.

Xylem anatomical differences existed between the two genotypes (Figs 8 and 9). There were no observed changes in xylem vessel diameter in PT between taproot and basal stem while CM presented larger vessel diameter in basal stem than in taproot (Fig 8A). Despite this increasing diameter in the CM basal stem, xylem vessel diameter was larger in PT than in CM, both in taproot and basal stem.

On the other hand, xylem vessel density was higher in the basal stem than in the taproot in both genotypes although this difference was greater in CM (Fig 8B). In CM, the increased vessel diameter and vessel density in basal stem compared to their values in taproot resulted in a total vessel lumen area 2.17-fold higher in basal stem than in taproot. PT showed a 1.22-fold higher total vessel lumen area in basal stem compared to that of taproot (Fig 8C).

## Discussion

The main objective of this work was to determine the influence of hydraulic conductance on transpiration in citrus. Apparently, transpiration rate (*E*) was not regulated solely by the degree of stomatal opening (Fig 2). However, results showed a linear relationship, identical for both studied genotypes (*P*. *trifoliata* [PT] and Cleopatra mandarin [CM]), between whole plant transpiration (*T*_p_) and plant hydraulic conductance (*K*_p_) (Fig 7).

Leaf biomass and Leaf dry weight/Root dry weight ratio (L/R) were higher in CM than PT. Both the transpiration rate (*E*) and the average root hydraulic conductance (*K*_r_) were higher in PT than in CM (Figs 2 and 3). However, CM presented higher average shoot hydraulic conductance (*K*_s_) value (Fig 3).

In both genotypes *K*_s_ and *K*_r_ were related positively with foliar and root biomass, respectively, as reported by other authors (e.g., [45]). When both parameters are scaled by dividing between leaf surface to obtain leaf-specific root hydraulic conductance (*K*_r-l_) and leaf-specific shoot hydraulic conductance (*K*_s-l_) (Table 2), *K*_r-l_ and *K*_*s-l*_ values were higher in PT. Both could determine the higher transpiration rate, stomatal conductance and net CO_2_ assimilation that PT presents, compared with CM [39, 40, 46].

**Table 2:** Root-specific root hydraulic conductance (*K*_r-r_), leaf-specific root hydraulic conductance (*K*_r-l_) and leaf-specific shoot hydraulic conductance (*K*_s-l_) in *P*. *trifoliata* (PT) and Cleopatra mandarin (CM).

	*K*_r-r_	*K*_r-l_	*K*_s-l_
	(10^−7^ x Kg MPa^-1^ s^-1^ g^-1^)	10^−5^ x Kg MPa^-1^ s^-1^ cm^-2^)	10^−5^ x Kg MPa^-1^ s^-1^ cm^-2^)
PT	28.12 ± 2.23 a	177.58 ± 9.18 a	66.80 ± 7.10 a
CM	14.37 ± 2.06 b	23.93 ± 3.01 b	24.44 ± 2.31 b

For each parameter, means (n = 16) with different letters show statistically significant differences (P<0.05).

A linear relationship between *K*_r_ and *K*_s_ was found in both genotypes, although this relationship was not the same for both types of plants. For equal *K*_r_ values, CM presented higher *K*_s_ than PT (Fig 4). Similar relationships between different plant hydraulic parameters have been described by other authors. [47] showed a linear regression between leaf hydraulic conductance and whole plant hydraulic conductance while [21] reported relationships between whole-plant and shoot hydraulic conductance. In our study, interestingly, for an individual plant independent of its genotype, *K*_s_ was always similar or lower than *K*_r_. Also [48] observed that whole leaf hydraulic conductance was lower than *K*_r_ in nineteen grass species. This could be an evolutionary trait to avoid cavitation. If *K*_s_ was greater than *K*_r_, and water flow in shoots was close to its possible peak flow, roots could not provide all the water claimed by the air component. This could result in increased water tension that would facilitate air bubble formation in the xylem. The lack of literature integrating the study of whole root and shoot systems in tree species does not enable us to pose this hypothesis. However, our results support the assumption that the design of plant hydraulic conductance and resistance to cavitation within a plant is optimized to deal with the conflicting balance between evaporative demand and protection from hydraulic failure [49].

The higher *K*_s_/*K*_r_ ratio in CM could be attributed to the anatomical features of the xylem of the main root and stem base. In CM seedlings, values of lumen diameter and density of vessels in the base of the stem were 27.9% and 32.4% higher compared with the values of these parameters in the taproot, resulting in a two-fold increase in total lumen vessel area compared with this area in the taproot, while in PT this increase was much lower (Fig 8). Therefore, the same as with xylem traits and L/R ratio, the *K*_s_/*K*_r_ ratio could be genetically determined. On the other hand, the higher diameter in xylem vessels in PT could be attributed to higher aquaporin activity. According to [50], aquaporins play a role in mediating water transport to support xylogenesis because plants with higher aquaporin expression achieve a greater final cell diameter in xylem vessels. In other studies, we have observed more aquaporin expression in roots of PT than in CM [24, 51], which could be partially responsible for the high root hydraulic conductance of PT plants, and also for their greater vessel diameter.

Clearly root and shoot growth must be coordinated somehow during the life of a plant [52]. The relationship between both *K*_r_ and *K*_s_ with plant biomass (Fig 5) and the relationship between both conductances (Fig 4) suggests that hydraulic traits should influence Shoot/Root ratio and plant growth, in accordance with other authors [36, 53, 54, 55]. It is known that growth of the aerial component in olive trees is related to *K*_r_ [53] and also that *K*_r_ is responsible for the vigor of grafted peach varieties [54]. However, although many studies associate high values of *K*_r-l_ or *K*_r-r_ (leaf or root-specific root hydraulic conductance) with strong shoot development, in our experiment CM showed a higher L/R ratio even though PT presented higher *K*_r-l_ and *K*_r-r_ (Table 2). Therefore, root hydraulic conductance may apparently determine the gas exchange features of leaves rather than shoot growth rate. [54] observed that non-vigorous peach varieties grafted on vigorous rootstocks presented lower *K*_s_, and less vigorous development than vigorous varieties grafted on the same rootstocks, indicating that genetic factors probably determine the *K*_s_ and growth of these varieties. Therefore, these could explain the higher L/R ratio in CM. Accordingly, the effect of rootstocks hydraulic traits on shoot growth would be more evident when rootstocks were grafted with same variety. Taking all this into account, in our study, the highest L/R ratio in CM could be more closely related with the *K*_s_/*K*_r_ ratio and xylem anatomy traits than with differences in water transport capacity of the root system.

On the other hand, *E* stayed practically constant between 10:00 am– 4:00 pm, even though stomatal closure was observed after 11:00 am. The reduction in *g*_s_ was probably due to the high temperature increase in late morning, which resulted in a high *VPD* (Fig 2D). By reducing *g*_s_, plants minimize water loss and maintain plant cell hydration as *VPD* increases. Despite reduced stomatal conductance in response to increasing vapor pressure deficit, transpiration rate can increase [56, 57], as occurred in this experiment, since *E*/*g*_s_ ratio increased (Fig 2C). This response varies among species and genotypes [58, 59, 60]. Our results showed that at early morning (from 8:00 am to 10:00 am *E* was limited by *VPD* and not by *g*_s_ (Fig 2A, 2B and 2D), which presented its maximum values in this period and was not completely related to the increasing *E*. At mid-range values of *VPD* (approximately from 10:00 am to 12:00 am, stomata progressively closed as *VPD* increased. However, *E* remained constant as the increase in the driving force (*VPD*) compensated the reduction in *g*_s_. It suggested that hydraulic limitation more than stomatal limitation were related to the maximum *E* values reached at midday. A number of studies have indicated a functional relationship between *g*_s_ and hydraulic conductance [18, 52]. Accordingly, as *VPD* increased, the rate of stomatal closure (which controls water losses) could be modulated by the plant hydraulic system (which controls water supply to the leaves). Therefore *E* would be the result of the balance between *g*_s_, *VPD* and hydraulic conductance.

The relationship between whole plant transpiration (*T*_p_) and *K*_r_, presented in Fig 6, has also been reported in citrus by [38]. The latter authors found a significant correlation (r^2^ = 0.63) between these two parameters, noting that rootstocks with low *K*_r_ values showed lower *T*_p_ values than rootstocks with higher *K*_r_ values. However, our study shows that apparently *K*_s_ also influences plant transpiration (Fig 6B). Previous studies on citrus rootstocks suggest that differences in the root size or in the crown volume of grafted varieties affect the plant-soil-water relationship [61, 62, 63, 64, 65]. Moreover, *T*_p_ in both genotypes were strongly associated with foliar biomass (Fig 1). This suggests that foliar biomass is probably an important factor determining transpiration, which in turn appears to be related to the fact that increases in foliar biomass are related to *K*_s_ increments (Fig 5B). Our results support the dependence of *T*_p_ on foliar biomass, in accordance with previous studies in citrus suggesting that the crown size influences the rate of water absorbed by trees grafted on different rootstocks [61] but also indicate that this is associated with the higher hydraulic conductance values reached with higher crown size.

The relationships between *K*_r_ and *K*_s_ with *T*_p_ differed between CM and PT (Fig 6), but the relationship between whole plant hydraulic conductance (*K*_p_) and *T*_p_ was identical for both genotypes (Fig 7). Shoot and root contribution to whole plant hydraulic conductance differed greatly between the two genotypes, even so, the relationship between *K*_p_ and *T*_p_ remained constant, suggesting that plant transpiration is regulated directly by *K*_p_, which in turn depends on *K*_r_ and *K*_s_ [10, 66]. Several studies have highlighted the coordination between plant water transport capacity and leaf-level gas exchange or photosynthetic capacity [67, 68, 69, 70, 71]. It is therefore assumed that a high plant hydraulic efficiency (i.e. high leaf-specific hydraulic conductance) which modulates transpiration is an essential prerequisite for a high productivity in tree species [3]. However, a decrease in future precipitation and an increase in the temperature have been predicted for many *Citrus* growing regions, affecting the plant water relations and potentially constraining its productivity [72]. Both CM and PT are widely used as citrus rootstocks. The ability of the rootstock to supply water and nutrients to the plant is a main factor influencing fruit development in citrus trees, determining the strength of the grafted variety and tolerance to water stress [73, 74]. When comparing grafted varieties onto CM and PT rootstocks, grafted varieties on CM presented less net CO_2_ assimilation, transpiration rate and reduced stomatal conductance than when are grafted in PT [23, 24]. However, grafted varieties on CM present higher tolerance to water stress probably attributed to the relatively low values of *K*_r-r and_ *K*_r-l_ that limits sap flow from roots to leaves promoting conservative water use and favoring better water balance in the plant [24, 75]. Therefore, in areas where water availability can be a limiting factor the use of CM could be a strategy for reducing water stress.

This work shows the need for integration of whole plant hydraulic system when attempting to understand the plant water relations and we conclude that transpiration in citrus is strongly determined by the hydraulic system of the whole plant and the differences in root and shoot contribution to *K*_p_ could be related to the leaf/root ratio, as well as to xylem anatomy characteristics.

## Supporting Information

S1 Table Number of leaves, dry weight of stem, leaves and root, leave surface and shoot, root and plant hydraulic conductance of the sixteen plants of Cleopatra Mandarin and Poncirus trifoliata used in the experiment. (XLSX)

S2 Table Stomatal conductance (*g*_s_), transpiration (*E*), *E*/*g*_s_ ratio and vapor pressure deficit (*VPD*) in *P*. *trifoliata* (PT) and Cleopatra mandarin (CM). At each hour of the day, values are means of six replicates for each type of plant.
(XLSX)

S3 Table Histological data correspond to the mean of six independent plants of *Poncirus trifoliata* and six independent plants of Cleopatra mandarin. (XLSX)
